# Cannabidiol for the Treatment of Cervical Spondyloarthritis-Related Pain: A Case Report

**DOI:** 10.7759/cureus.67224

**Published:** 2024-08-19

**Authors:** Valdecir C Tadei

**Affiliations:** 1 Anesthesiology, Faculdade de Medicina de São José do Rio Preto, São José do Rio Preto, BRA

**Keywords:** spondyloarthritis, pain management, chronic pain, cannabis medicine, prescribed cannabis

## Abstract

Spondyloarthritis (SA) is a chronic inflammatory disease that predominantly affects the spinal column. SA-related pain can be intense, persistent, and disabling. Studies with cannabis have been conducted involving patients with refractory epilepsy, multiple sclerosis, Parkinson’s disease, sleep disorders, and chronic pain. Cannabidiol is the major non-psychotropic component of cannabis, has anti-inflammatory and analgesic properties, and exerts anxiolytic and mood-stabilizing effects. This paper reports a case of a 72-year-old male with SA, with mild stenoses of the spinal canal at C4-C5 and C5-C6 and stenoses of the left neural foramina at C3-C4, C4-C5, C5-C6, and C6-C7. The use of cannabidiol in our patient achieved satisfactory results in the control of pain related to cervical spondyloarthritis.

## Introduction

Spondyloarthritis (SA) is a chronic inflammatory disease that predominantly affects the spinal column. SA generally begins in young adulthood and is associated with accentuated physical impairment and a significant reduction in quality of life [1]. SA-related pain can be intense, persistent, and disabling [2].

The treatment of patients with chronic pain can be a therapeutic challenge for physicians, as it consists of the use of analgesics and physiotherapy, with variable responses and short-lasting results [3]. Cannabinoids are relevant in this context, as these substances affect all pain pathways responsible for the transmission, modulation, or perception of pain [4,5]. Cannabidiol (CBD) is a cannabinoid that has significant analgesic, anti-inflammatory, anticonvulsant, and anxiolytic properties that can be useful in the treatment of patients with chronic pain and is considered relatively safe, with few side effects [6-8].

This paper describes a case of the use of cannabidiol as a therapeutic option for pain related to cervical spondyloarthritis and improvement in quality of life.

## Case presentation

**Figure 1:**
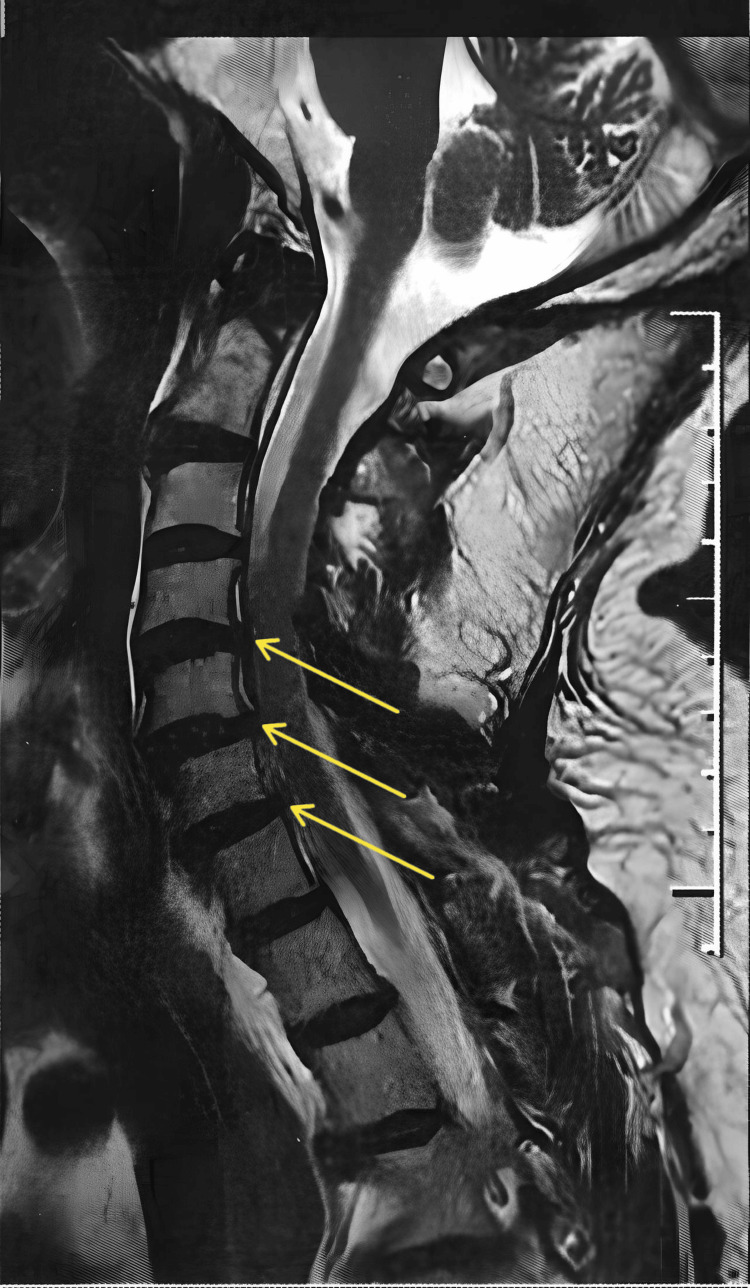
Magnetic resonance imaging of the cervical spine showing spondyloarthrosis, highlighting mild stenosis of the spinal canal at C4-C5 and C5-C6 and stenoses of the left neural foramina at C3-C4, C4-C5, C5-C6, and C6-C7

A 72-year-old, white, male, diabetic, hypertensive, and retired physician with spondyloarthritis and mild stenoses of the spinal canal at C4-C5 and C5-C6 and stenoses of the left neural foramina at C3-C4, C4-C5, C5-C6, and C6-C7 (Figure 1) presented with constant neuropathic pain in the cervical spine and left shoulder and arm for more than one year, numbness on the left side of the face, diminished muscle strength, and pain intensity of 8 on the visual analog scale (VAS) of 1 to 10.

Electroneuromyography for an analysis of upper limb pain showed a chronic neurogenic pattern in muscles corresponding to the myotomes on the right at C7 and the left at C7/T1, without acute denervation, compatible with cervical radiculopathy on the right at C7 and the left at C7/TI, moderate on the right and severe on the left. The patient made continual use of olmesartan 40 mg + amlodipine besylate 5 mg, dapaglifosin 10 mg, analgesics (tramadol, dipyrone, ibuprofen), and physiotherapy sessions (a duration of 3 weeks without clinical improvement). Physical activity (karate, walking, and swimming) was interrupted and labor was not possible due to pain, leading to retirement.

Treatment involved full-spectrum medicinal cannabis oil 6000 mg (200 mg of CBD/mL and 0.3% tetrahydrocannabinol). The dose began at 8 mg every 12 h, with an increase every four days to 100 mg every 12 h. Signs of improvement emerged after 20 days of use (approximate dose of 50 mg every 12 h). After 90 days of treatment, the patient reported the absence of pain, the return of physical activity (walking and swimming), and the suspension of the use of analgesics. Muscle strength increased considerably and tremors in the left arm had diminished by 90%. The transient side effect was sleepiness only at the onset of treatment. The patient has been taking cannabidiol for one year, with adjustments to lower the dose (currently 144 mg a day).

## Discussion

Chronic pain exerts an enormous personal and economic impact, affecting more than 30% of individuals throughout the world [9]. According to the Centers for Disease Control and Prevention (CDC), approximately 20.4% of adults in the United States had chronic pain in 2019 [10].

Various studies have been conducted on the use of cannabis as a therapeutic option in cases of chronic pain. Current standard treatment involves opioids, which have side effects such as severe constipation, nausea, sleepiness, respiratory depression, and opiate dependence [11]. In this case, the patient took an opioid continually but suspended the use of all analgesics, including the opioid, after 90 days of treatment with CBD due to the absence of pain. The only side effect reported by the patient was mild sleepiness at the onset of treatment.

Treatment with CBD should be based on the control of pain and consequent improvement in quality of life. In the present case, the pain was relieved, the opioid was suspended, and the patient returned to the practice of physical activity, all of which contributed to an improvement in quality of life. This clinical outcome indicates that the therapeutic conduct was adequate, which is in agreement with the findings described in the literature. In a longitudinal prospective study involving 751 patients with chronic pain, Safakish et al. investigated the effects of medicinal cannabis and found significant improvements in both pain and health-related quality of life [12].

Randomized, placebo-controlled, double-blind, clinical trials are needed to prove the effectiveness of CBD in patients with spondyloarthritis-related pain, including different lines of CBD-enriched Cannabis extracts, different doses, and different lengths of treatment to investigate the effectiveness, safety, and tolerance.

## Conclusions

The use of cannabidiol in our patient achieved satisfactory control of pain related to cervical spondyloarthritis. Based on this result and considering scientific evidence of the effectiveness of CBD in the treatment of patients with chronic pain due to different illnesses, this therapeutic option may be beneficial to such patients when conventional medicinal treatment is unsatisfactory. However, clinical trials are needed to confirm the effectiveness of CBD in patients with spondyloarthritis-related pain.
